# Predicted and final tooth position assessment following indirect bonding planned by a digital system

**DOI:** 10.1590/2177-6709.30.1.e252451.oar

**Published:** 2025-04-07

**Authors:** Fernando César MOREIRA, Helder Baldi JACOB, Guilherme dos Reis Pereira JANSON, Daniela Gamba GARIB

**Affiliations:** 1 Instituto de Pesquisas Energéticas e Nucleares (IPEN), Centro de Lasers e Aplicações (São Paulo/SP, Brazil).; 2 University of Oklahoma Health Sciences College of Dentistry, Department of Developmental Sciences, Division of Orthodontics (Oklahoma, USA).; 3 Faculdade de Odontologia da Universidade de São Paulo, Departamento de Ortodontia (Bauru/SP, Brazil).; 4 Faculdade de Odontologia da Universidade de São Paulo (Bauru/SP, Brazil).

**Keywords:** Dental models, Dental technology, Orthodontic tooth movement, Dental occlusions, Artificial intelligence, Modelos dentários, Tecnologia dentária, Movimentação dentária ortodôntica, Oclusões dentárias, Inteligência artificial

## Abstract

**Introduction:**

The purpose of this study was to evaluate the agreement between the predicted and the achieved tooth position planned by an orthodontic digital system.

**Methods:**

Digital models of the setup (Predicted) and the treated (Treated) groups of 23 subjects with Class I malocclusion were obtained. Digital models (Predicted and Treated) of each patient were superimposed, and referential geometric planes were constructed for linear and angular measurements: arch perimeter, arch depth, intercanine and intermolar widths, mesiodistal crown angulation, and buccolingual crown inclination. Bland-Altman analysis was performed to establish the agreement between the measurements. Spearman’s correlation coefficient was used to evaluate the correlation between groups.

**Results:**

Compared to Predicted group, the Treated group presented larger linear measurements for all measurements: 1) arch perimeter: 1.77±2.10 mm (maxilla) and 1.78±1.74 mm (mandible); 2) arch depth: 0.50±0.69 mm (maxilla) and 0.38±0.81 mm (mandible); 3) intercanine width: 0.30±0.98 mm (maxilla) and 0.49±0.64 mm (mandible), and; 4) intermolar width: 0.70±1.63 mm (maxilla) and 1.13±1.62 mm (mandible). Seven out of 14 angular measurements showed statistical differences between Predicted and Treated groups in the maxilla, while six out of 14 angular measurements were statistically significant between the two groups; the differences ranging from -8.91º to 1.91º and from -3.53° to 9.59° in the maxilla and mandible, respectively.

**Conclusions:**

The agreement between the Predicted and Treated groups was majority within the limits. The predictions of the digital system were not accurate in some parameters; however, most of the differences were within clinical acceptable range. Although there are some inaccuracies, the limitations do not seem to interfere with clinical outcomes and the quality of the treatment.

## RESUMO

**Objetivo:**

O objetivo deste estudo foi avaliar a concordância entre a posição dentária preditiva e a obtida, conforme planejado por um sistema ortodôntico digital.

**Métodos:**

Foram obtidos modelos digitais dos grupos no *setup* (Preditivo) e após o tratamento (Tratado) de 23 indivíduos com má oclusão de Classe I. Os modelos digitais (preditivos e tratados) de cada paciente foram sobrepostos, e planos geométricos referenciais foram construídos para obter as seguintes medidas lineares e angulares: perímetro da arcada, profundidade da arcada, distâncias intercaninos e intermolares, angulação mesiodistal da coroa e inclinação vestibulolingual da coroa. A análise de Bland-Altman foi realizada para estabelecer a concordância entre as medidas. O coeficiente de correlação de Spearman foi utilizado para avaliar a correlação entre os grupos.

**Resultados:**

Comparado ao grupo Preditivo, o grupo Tratado apresentou medidas lineares maiores para todas as medidas: 1) perímetro da arcada: 1,77±2,10 mm (maxila) e 1,78±1,74 mm (mandíbula); 2) profundidade da arcada: 0,50±0,69 mm (maxila) e 0,38±0,81 mm (mandíbula); 3) distância intercaninos: 0,30±0,98 mm (maxila) e 0,49±0,64 mm (mandíbula); e 4) distância intermolares: 0,70±1,63 mm (maxila) e 1,13±1,62 mm (mandíbula). Entre as quatorze medidas angulares, sete mostraram diferenças estatísticas entre os grupos Preditivo e Tratado na maxila, enquanto seis das quatorze medidas angulares foram estatisticamente significativas entre os dois grupos; as diferenças variaram de -8,91º a 1,91º e de -3,53° a 9,59° na maxila e mandíbula, respectivamente.

**Conclusões:**

A concordância entre os grupos Preditivo e Tratado foi majoritariamente dentro dos limites. As previsões do sistema digital não foram precisas em alguns parâmetros; no entanto, em sua maioria as diferenças estavam dentro da faixa clinicamente aceitável. Embora existam algumas imprecisões, as limitações não parecem interferir nos resultados clínicos e na qualidade do tratamento.

## INTRODUCTION

The indirect bonding technique has been associated with the orthodontic digital system. With the assistance of software programs, clinicians can digitally position brackets precisely, view possible occlusal interferences, and predict the occlusion before bonding,^1^ increasing effectiveness and efficiency in the clinic.^2^ ^,^ ^3^ The digital placement of the bracket with the aid of computer-aided design and manufacturing (CAD-CAM) allows orthodontic treatment to reduce the number of appointments and chair time.^1^ ^,^ ^2^ In addition, the digital system supports the use of orthodontic virtual setups to predict final teeth alignment and leveling.^1^ ^,^ ^4^

The correct position of the teeth in the dental arch favors a balanced and stable occlusion. For this reason, it is important that the orthodontic setup determines the best position of the teeth in the three planes of space, in order to properly correct the malocclusion and calculate the possibilities, according to the limitations of each particular case. Evaluating the potentialities and efficacy of the CAD-CAM system, a previous study, which used the American Board of Orthodontics objective grading system (ABO-OGS) for scoring dental casts and panoramic radiographs,^5^ found the digital system closely predicts the final teeth alignment and leveling.^1^ Contradictorily, significant discrepancies between simulated and final tooth positions were reported in a study evaluating the accuracy of a CAD-CAM-based lingual orthodontic treatment.^6^

Few studies have compared the accuracy of the predicted occlusion provided by the CAD-CAM system and the final occlusion after the orthodontic therapy using digital measurement of angulation and linear tooth movements. Although slightly larger on treated occlusion than on virtual setup, measurements from virtual setup and treated occlusion produce accurate representations.^1^ In addition, if an individual is adequately calibrated, measures from virtual setup and treated occlusion produce accurate and reliable outcomes.^1^ A retrospective cohort study evaluated the efficacy and efficiency of two CAD-CAM systems, showing that the lingual bracket system is more effective than the labial system bracket, when the two treatment systems were critically compared.^7^ Other studies compared CAD-CAM predicted occlusion to the final treatment occlusion using the 3-dimensional (3D) model superimposition method, showing small differences for most teeth.^1^ ^,^ ^8^ ^,^ ^9^

Thus, the purpose of the present study was to evaluate the agreement between the predicted and the achieved tooth positions planned by an orthodontic digital system. Measurements of linear and angular tooth positions were used to compare linear arch dimensions, tooth inclinations and angulations between predicted occlusion and final occlusion achieved from 3D models of subjects with Class I malocclusion treated without tooth extractions. The null hypothesis was that there would be no difference between the tooth positions and arch dimensions as predicted using a CAD-CAM system and the orthodontic treatment.

## MATERIAL AND METHODS

### ETHICAL APPROVAL AND ELIGIBILITY CRITERIA

This retrospective study was approved by the ethical committee of the Bauru School of Dentistry of the University of São Paulo, under protocol number 4.552.088. The samples were obtained from a previous study by Moreira et al.^1^, and comprised 23 patients who presented Angle Class I malocclusion with mild crowding or spacing and not requiring orthodontic tooth extraction. The requirements for patient inclusion were: 1) complete permanent dentition (excluding third molars); 2) Angle Class I molar relationship on both sides of the dental arch; 3) Angle Class I canine relationship or less than ¼ cusp in Class II; 4) slight or absent dental posterior crossbite. The exclusion criteria were as follows: 1) caries lesions that compromise the dental structure; 2) morphologic variation in size and shape of the crown; 3) open bite or deep bite equal to or greater than 4 mm; and 4) crowding or tooth spacing greater than 4 mm.

### PATIENTS AND ORTHODONTIC DIGITAL WORKFLOW

The patients received orthodontic treatment planned by CAD-CAM technology associated with the indirect bracket bonding method. Computer algorithm determined the planned bracket position on the buccal surface of the teeth in the 3D model. Initially, technicians carried out the treatment plan after receiving the digitized models obtained by alginate impressions of the arches of all patients. An experienced orthodontist (FCM) revised, adjusted, and approved the final teeth positions and occlusion before the indirect trays production. The system created a 3D model with pads on each tooth surface, to guide the correct placement of the bracket in the ideal planned position. EasyClip Plus 0.022 x 0.028-in self-ligating brackets (Aditek Orthodontics, Cravinhos, São Paulo, Brazil) were positioned on each pad of the respective tooth on the 3D-printed model. Transparent and flexible indirect bond thermoformed trays were manufactured using Biolon^™^ and Drufolen^™^ H (Dreve Dentamid GmbH, Unna, Germany).^3^

After the initial indirect bonding, Damon archwires (Ormco, Orange, CA, USA) were used for alignment and leveling, according to the following sequence: maxillary and mandibular 0.014-in, 0.018-in, 0.017 x 0.025-in, 0.019 x 0.025-in nickel-titanium, and 0.019 x 0.025-in stainless steel wires coordinated on the dental arches diagram planned and sent by the CAD-CAM system. The 0.019 x 0.025-in stainless steel archwires were maintained for 3 months, to allow optimal movement of the teeth programmed by the system according to the bracket position. No wire bending and/or inter or intramaxillary elastic was used during orthodontic treatment, to avoid interference with the outcome programmed by the software. In addition, interproximal tooth reduction was not performed in any of the patients, to avoid dimensional changes in the dental arches. 

After final alignment and leveling, traditional plaster models were obtained with alginate impressions of the dental arches; the 3D models from the plaster casts of the patients were acquire by digitization using a 3Shape R700 desktop scanner (3Shape Dental System, Copenhagen, Denmark). The reconstruction of the digital models was obtained by 3Shape ScanIt^®^ software, producing files of the type 3Shape zip format (.3sz). The files were exported to Ortho Analyzer^™^ 2013 software and converted to stereolithography (.stl file format). Then, all digital models were exported to the platform eXceed^™^, to create the virtual setups. 

**Figure 1:**
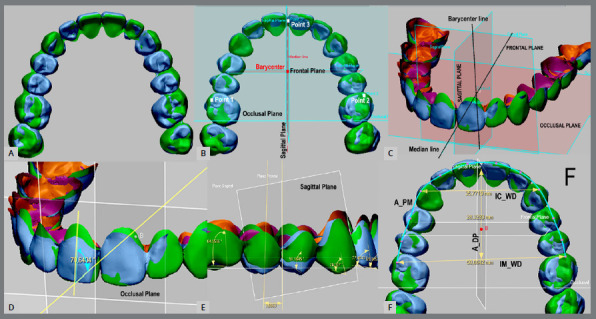
Global alignment of the Predicted and Treated 3D group models (A), followed by superimposition by iterative closest point algorithm method (B). Occlusal, Frontal and Sagittal reference planes were built, to standardize measurements (**C, D,** E). Arches measurements (F): arch perimeter ( A_PM ), arch depth ( A_DP ), intercanine width ( IC_WD ) and intermolar width ( IM_WD ).

Predicted and treated digital dental 3D models were created for 23 patients. To calculate the sample power, a *post-hoc* test was used. The effect size of Cohen’s d was calculated (0.70) to obtain a test power of 0.64 with a significance level of 5%. Three-dimensional digital models were obtained according to the following steps:

1. Acquisition of the 3D models: The Predicted group presented the eXceed^™^ virtual setup of each patient, obtained by exporting initial 3D models via web to Doctor WebGL software (eXceed, Witten, Germany), after careful evaluation and approval of the treatment. These digital models were obtained by scanning maxillary and mandibular dental plaster models using a desktop scanner (3Shape, Copenhagen, Denmark). ScanIt software tools (3Shape) performed the reconstruction and preparation of all the digital 3D models. Then, all produced data files (.3sz) were exported to Ortho Analyzer 2013 software (3Shape, Copenhagen, Denmark), for conversion to stereolithography (.stl file format), and they were exported to the company to create setups. Treated group presented the 3D models after the orthodontic alignment and leveling phase (0.019 x 0.025-in stainless steel archwire for 3 months).
2. 3D model data refinement: The standard tessellation files obtained were exported to Geomagic Design X software (3D Systems, Rock Hill, SC) to trim and repair small defects in the mesh of the 3D models, using polygons tools of the software. In addition, all brackets and tubes were digitally removed from the buccal surface of the teeth, using the poly-face removal tool and smart repair of selected holes of the Geomagic Design X software. Data files were exported into Geomagic Control X software (3D Systems, Rock Hill, SC) to set landmarks on each tooth and to create reference planes to standardize measurements.
3. 3D model superimposition: Predicted and treated 3D models of each respective dental arch of all patients were globally aligned following superimposition, by choosing three landmarks on the buccal surface of the right central incisor, right first molar, and left first molar. Although treated scanned 3D dental models of the maxilla are reliable to superimposition method, predicted 3D models provided by the CAD-CAM system presented no palatal rugae, so no reliable stable anatomical structure was common between the 3D models of the Predicted and Treated groups. Accordingly, the refinement of the alignment of the matched digital models was achieved using a best fit tool within the software, which involves an iterative closest point algorithm (Fig. 1).
4. Reference planes: The last step was the building of the reference planes to establish the known coordinates of both groups in the three-dimensional space of the software. Three geometric planes (1/100 mm scale) were constructed on each 3D model of all subjects, and the intersection among them formed three 90-degree anatomical reference axes. The coordinates and reference planes were created according to Sjögren et al.^10^ (Fig. 1).

### MEASUREMENTS PARAMETERS

Linear variables were measured as follows: (1) arch perimeter (A_PM) - the distance from the mesial surface of the first molar around the dental arch to the same point in the opposite side; (2) arch depth (A_DP) - the perpendicular distance from the first permanent molars to the incisors; (3) intercanine width (IC_WD) - the distance between the cusp tips of the right and left canines; and (4) intermolar width (IM_WD) - the distance between the mesiobuccal cusp tips of the right and left first molars. The following angular variables were measured: (5) mesiodistal crown angulation (MD), and (6) buccolingual crown inclination (BL). Crown mesiodistal angulations were measured between the long axis of the crown and the sagittal or frontal plane of the posterior and anterior teeth, respectively. Crown buccolingual inclinations were determined by the resulting angle between the tooth long axis and the occlusal plane (Fig. 1). One calibrated observer (FCM) performed all angular and linear measurements. The threshold values for comparative analysis to accuracy were set at 0.50 mm and 2° for linear and angular dimensions, respectively. These threshold values were selected as they represent accepted professional standards during case evaluation using the ABO-OGS.^11^

### STATISTICAL ANALYSIS

Data analyses were performed using Microsoft Excel 2013 (Microsoft Corp., Redmond, WA) and GraphPad Prism 9 (GraphPad Software, San Diego, CA). The homogeneity of variance and normality of the residuals were established by the Shapiro-Wilk, and D’Agostino-Pearson normality tests, respectively. Intraobserver random error was estimated using Dahlberg method errors and intraclass correlation coefficients (ICC).^12^ Intraobserver systematic errors between the replicates were described as mean differences and compared statistically with paired *t* tests. Descriptive statistics and paired *t* tests were carried out for all six parameters evaluated. Bland-Altman analysis was used to establish the reliability of all measurements between both groups (Predicted and Treated) and to verify the accuracy of the CAD-CAM system at the end of alignment and leveling. Spearman’s correlation coefficient was calculated to evaluate the correlation between predicted and actual tooth positions after alignment and leveling. All statistical analysis were performed using a significance level of 5% (α=0.05).

## RESULTS

The samples comprised 23 patients (15 women and 8 men, averaging 26.14 ± 6.53 years of age). The average treatment time was 20.79 ± 2.55 months at the end of alignment and leveling using the planned arch provided by the orthodontic system. The data obtained showed that there was no statistical significance between the right and left homologous maxillary and mandibular teeth (probability ranging from 0.054 to 0.996 and from 0.064 to 0.817, respectively). Due to similar pattern trends, the right and left sides of the 3D models were grouped to carry out statistical comparisons between Predicted and Treated groups.

### INTRAOBSERVER SYSTEMATIC AND INTRAOBSERVER RANDOM ERROR

**Figure 2:**
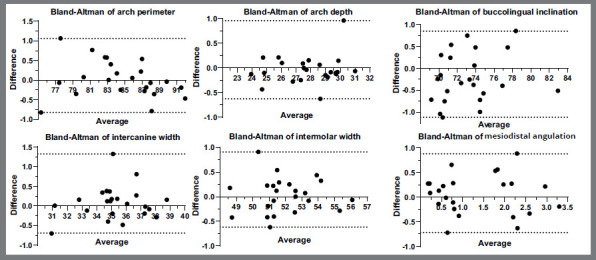
Bland-Altman analysis plots for intraobserver reliability. Repeatability of the six measurements: arch perimeter, arch depth, intercanine width and intermolar width, mesiodistal angulation and buccolingual inclination.

Intraobserver systematic errors of the replicates showed similar values (Table 1). The method errors ranged from 0.20 mm to 0.32 mm and 0.29° to 0.43° in the linear and angular measures, respectively, and the intraclass correlation coefficient ranged from 0.914 to 0.995, showing a high degree of reliability (Table 2). Bland-Altman plot analysis showed that the differences between replicated measurements of the six criteria were within acceptable values, but two outliers showed differences ranging up to approximately 1.00 mm (Fig. 2).

**Table 1:** Intraobserver systematic errors of the six variables among replicate measurements. Systematic differences (mm) were calculated and the probability were estimated by paired t tests statistical analysis. Measurements were based on maxillary arches and maxillary central incisors.

Variables	Mean (1^st^)	SD	Mean (2^nd^)	SD	Diff	SD	Prob.
Arch perimeter (mm)	84.55	4.46	84.52	4.58	-0.02	0.47	0.797
Arch length (mm)	27.79	2.05	27.82	2.00	0.03	0.29	0.593
Intercanine width (mm)	35.31	2.01	35.23	1.95	-0.08	0.42	0.339
Molar width (mm)	52.01	1.82	51.97	1.82	-0.03	0.35	0.650
Buccolingual inclination (degrees)	73.10	3.39	73.32	3.27	0.21	0.58	0.098
Mesiodistal angulation (degrees)	1.34	1.00	1.30	0.99	-0.06	0.40	0.454

*Systematic differences (mm) were calculated and the probability was estimated by paired t tests statistical analysis (significance level at p<0.05).

**1^st^: first measurement; 2^nd^: second measurement; SD - Standard Deviation; Diff: Mean Difference; Prob: Probability.

**Table 2:** Descriptive statistics and intraobserver random errors between estimated measurements with method errors (mm) and intraclass correlation coefficient (ICC). Measurements were based on maxillary arch and maxillary central incisor.

Variables	Min-max (1st)	Min-max (2nd)	ME	RI (%)	ICC
Arch perimeter (mm)	74.91 - 91.74	75.74 - 92.20	0.32	99.45	0.995
Arch depth (mm)	23.88 - 30.97	24.01 - 31.03	0.20	98.92	0.989
Intercanine width (mm)	30.58 - 38.84	31.15 - 38.68	0.29	97.66	0.978
Molar width (mm)	48.51 - 56.04	48.51 - 56.11	0.24	98.04	0.980
Buccolingual inclination (degrees)	68.80- 82.67	69.52 - 83.18	0.43	98.24	0.967
Mesiodistal angulation (degrees)	0.05 - 3.20	0.04 - 3.39	0.29	91.25	0.914

*1^st^: first measurement; 2^nd^: second measurement; Min.: Minimum; Max.: Maximum; ME: Method Errors; RI: Reliability Index; ICC: Intraclass Correlation Coefficient.

### PREDICTED X TREATED GROUPS

**Figure 3:**
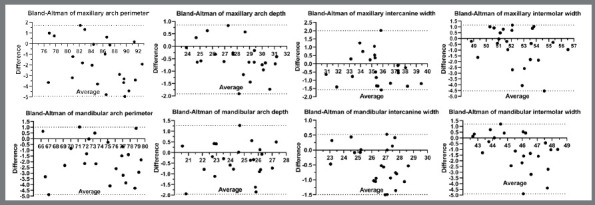
Bland-Altman plots of each four linear measurements of the maxillary and mandibular arches of the Predicted and Treated groups.

Linear dimensions: Bland-Altman plots of each of the four linear measurements (Fig. 3) indicated variances of -4.95 mm to 2.01 mm (maxillary arch) and -4.87 mm to 1.01 mm (mandibular arch). Maxillary and mandibular arches perimeters showed similar values and, according to the standardized threshold values of 0.50 mm and 2° for comparative analysis of accuracy for linear and angular dimensions, respectively, 20 out of 23 measurements (87%) were above the ABO threshold (0.50 mm).^11^ Maxillary and mandibular arch depth showed that 16 out of 23 measurements (69%) and 12 out of 23 (52%), respectively, were above the threshold values. Transversal dimensions of maxillary and mandibular arches showed that 16 out of 23 (69%) and 15 out of 23 (65%), respectively, to intercanine width; and 16 out of 23 (69%) to intermolar width in both arches were above the threshold value (Fig. 3).^11^

**Figure 4:**
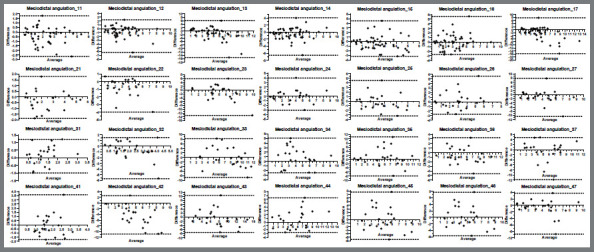
Bland-Altman plots of mesiodistal angulation means of all teeth of the maxillary and mandibular arches of the Predicted and Treated groups (international teeth numbering system).

**Figure 5:**
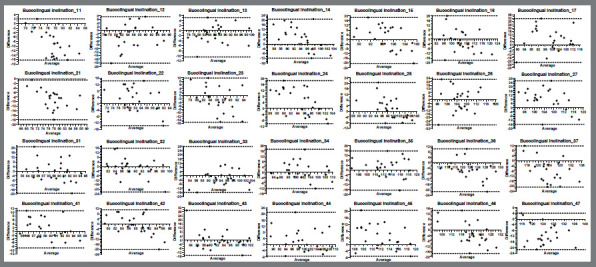
Bland-Altman plots of buccolingual inclination means of all teeth of the maxillary and mandibular arches of the Predicted and Treated groups (international teeth numbering system).

Angular dimensions: Bland-Altman plots of each two angular measurements indicated variances of -15.67º to 7.81º (maxillary arch) and -11.79º to 10.50º (mandibular arch) to angular variables means of all teeth (Fig. 4), and -28.08º to 31.55º (maxillary arch) and -22.20º to 29.21º (mandibular arch) to inclination variables means of all teeth (Fig. 5). A crown-torque and tip inadequacy of 2° causes a marginal ridge discrepancy of 0.5 mm in an average-sized molar.^11^ ^,^ ^13^ Maxillary and mandibular mesiodistal crown angulation showed that 63 out of 322 measurements (19%) and 98 out of 322 (30%), respectively, ​​were above the ABO threshold value (Fig. 4).^11^ ^,^ ^13^ Regarding buccolingual crown inclinations, Bland-Altman plots of maxillary and mandibular arches results showed that 272 out of 322 (84%) and 261 out of 322 (81%), respectively, were above the threshold of 2° (Fig. 5).^11^

**Figure 6:**
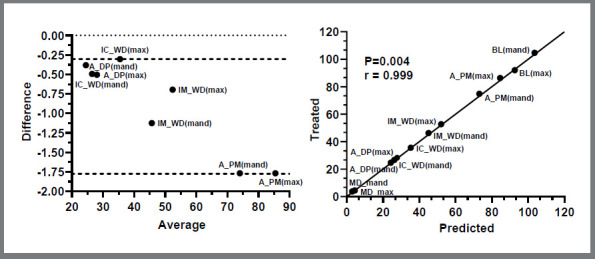
Bland-Altman plots of all grouped linear variables between two methods. Spearman’s correlation coefficient quantified linear and angular covariation between Predicted and Treated groups in both maxillary (Max) and mandibular (Mand) 3D models: arch perimeter (A_PM), arch depth (A_DP), intercanine width (IC_WD) and intermolar width (IM_WD), Mesiodistal angulation (MD) and buccolingual inclination (BL).

Spearman’s correlation coefficient was calculated to evaluate the correlation of the linear and angular dimensions between predicted and final tooth after the alignment and leveling in both maxillary and mandibular 3D models. The arithmetic mean of each linear and angular measurement was compared between all predicted and final occlusion (maxilla and mandibular), and high correlation between both methods was observed (Fig. 6). 

### MAXILLARY 3D MODEL COMPARISONS

Maxillary comparisons between the Predicted and Treated groups showed statistical significance in 50% of the measurements presented (Table 3). Arch perimeter and arch depth were significantly larger when measured on the Treated group digital model than Predicted group digital model (86.32 mm vs. 84.55 mm and 28.30 mm vs. 27.80 mm, respectively). There was a slight larger value in the transversal dimensions in the treated group compared to the setup for intercanine width (35.62 mm vs. 35.32 mm, respectively) and intermolar width (52.71 mm vs 52.01 mm, respectively). In regards to buccolingual crown inclination (Table 3), the Treated group showed significant smaller values than the Predicted group for the central incisors (8.21° vs. 17.13°), first premolars (-1.64° vs. -6.83°), and second molars (-8.01° vs. -15.80°). On the other hand, the Treated group showed significantly larger mesiodistal crown angulation than the Predicted group (Table 3) for the central incisors (2.37° vs. 1.99°), lateral incisors (3.26° vs. 2.55°), canines (5.64° vs. 4.44°), and second molars (5.52° vs. 3.61°).

**Table 3:** Maxillary measurement descriptive and comparison statistics for Predicted and Treated groups.

	Variables	Predicted occlusion		Treated occlusion		Treated - Predicted			
		Mean	SD	Mean	SD	Diff	SD	Prob.	Correlation coefficient
Linear (mm)	Arch perimeter	84.55	4.47	86.32	5.25	1.77	2.10	<0.001	0.918
	Arch depth	27.80	2.05	28.30	2.34	0.50	0.69	0.002	0.958
	Intercanine width	35.32	2.02	35.62	2.17	0.30	0.98	0.148	0.893
	Intermolar width	52.01	1.83	52.71	2.27	0.70	1.63	0.053	0.703
Buccolingual inclination (degrees)	Central incisor	17.13	3.34	8.21	5.64	-8.91	4.98	<0.001	0.482
	Lateral incisor	6.66	6.28	6.59	7.48	-0.06	7.93	0.954	0.346
	Canine	4.93	5.28	3.42	5.83	-1.50	5.53	0.071	0.507
	First premolar	-6.83	3.82	-1.64	8.13	-5.19	9.12	<0.001	-0.038
	Second premolar	-9.14	4.37	-7.04	8.29	-2.10	8.88	0.115	0.122
	First molar	-15.02	7.50	-15.62	9.02	0.59	11.25	0.720	0.083
	Second molar	-15.80	9.75	-8.01	11.09	-7.78	12.00	<0.001	0.342
Mesiodistal angulation (degrees)	Central incisor	1.99	1.25	2.37	1.38	0.38	0.94	0.008	0.749
	Lateral incisor	2.55	1.69	3.26	1.96	0.72	1.46	0.001	0.687
	Canine	4.44	2.38	5.64	3.71	1.20	3.71	0.032	0.322
	First premolar	3.31	2.06	3.70	1.99	0.38	1.73	0.141	0.634
	Second premolar	2.29	1.46	2.59	1.71	0.30	1.63	0.220	0.483
	First molar	2.97	2.07	2.93	1.72	-0.04	1.67	0.869	0.623
	Second molar	3.61	2.23	5.52	4.12	1.91	4.08	0.003	0.289

Bold indicates statistically significant differences at p < 0.05, 95% CI.

*SD - Standard Deviation; Diff: Mean Difference; Prob: Probability.

### MANDIBULAR 3D MODELS COMPARISONS

Mandibular arch measurement comparisons between Predicted and Treated group were statistically significant for 10 out of 18 analyzed parameters (Table 4), showing similar measures between anterior teeth and first premolars (Table 4). All four linear measurements were statistically significant and larger in the Treated group, when compared to the Predicted group (Table 4): arch perimeter (74.89 mm vs. 73.12 mm), arch depth (24.68 mm vs. 24.29 mm), intercanine width (26.73 mm vs. 26.24 mm), and intermolar width (46.24 mm vs. 45.11 mm). The buccolingual differences were registered only in the posterior teeth (Table 4). Second premolar presented a smaller buccolingual inclination in the Treated group than Predicted group (-20.72° and -24.25°, respectively), and the first and second molars showed significant more buccolingual crown inclination in the Treated group (-32.90° and -38.81°, respectively) than in the Predicted group (-28.89° and -29.21°, respectively). Regarding mesiodistal crown angulation (Table 4), differences between Predicted and Treated groups were statistically significant at the lateral incisor (0.65°), first premolar (0.87°), and first molar (0.59°). Lateral incisor and the first molar angulation were significantly larger in the Treated group than in the Predicted group (2.53° vs. 1.88° and 5.19° vs. 4.60°, respectively), and the first premolar angulation was significantly smaller in the Treated group than in the Predicted group (4.51° vs. 5.38°).

**Table 4:** Mandibular measurement descriptive and comparison statistics for Predicted and Treated groups.

	Variables	Predicted occlusion		Treated occlusion		Treated - Predicted			
		Mean	SD	Mean	SD	Diff	SD	Prob.	Correlation coefficient
Linear (mm)	Arch perimeter	73.12	4.19	74.89	4.34	1.78	1.74	<0.001	0.917
	Arch depth	24.29	1.95	24.68	1.91	0.38	0.81	0.033	0.913
	Intercanine width	26.24	1.43	26.73	1.66	0.49	0.64	0.001	0.926
	Intermolar width	45.11	1.49	46.24	2.20	1.13	1.62	0.003	0.680
Buccolingual inclination (degrees)	Central incisor	0.53	3.86	1.16	5.53	0.63	7.58	0.575	-0.264
	Lateral incisor	1.77	4.62	1.10	7.71	-0.66	8.16	0.581	0.201
	Canine	-3.40	6.23	-3.70	7.46	0.29	8.92	0.821	0.161
	First premolar	-11.66	5.39	-9.76	6.84	-1.88	6.94	0.071	0.350
	Second premolar	-24.25	4.46	-20.72	6.12	-3.53	7.31	0.002	0.070
	First molar	-28.89	4.26	-32.90	7.70	4.01	7.74	0.001	0.267
	Second molar	-29.21	5.33	-38.81	7.78	9.59	8.59	<0.001	0.181
Mesiodistal angulation (degrees)	Central incisor	1.58	0.86	1.53	0.78	-0.06	1.00	0.709	0.253
	Lateral incisor	1.88	1.07	2.53	1.61	0.65	1.69	0.012	0.254
	Canine	7.36	3.07	7.66	4.23	0.29	4.22	0.640	0.365
	First premolar	5.38	2.85	4.51	2.63	-0.87	2.34	0.014	0.638
	Second premolar	5.73	2.94	4.98	3.19	-0.75	3.80	0.185	0.231
	First molar	4.60	2.74	5.19	3.03	0.59	1.50	0.010	0.869
	Second molar	4.29	2.85	4.95	3.21	0.65	3.39	0.209	0.380

Bold indicates statistically significant differences at p < 0.05, 95% CI.

*SD - Standard Deviation; Diff: Mean Difference; Prob: Probability.

### BRACKET BOND FAILURE

**Figure 7:**
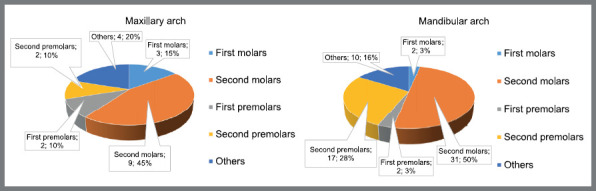
Distribution and percentage of brackets rebonding due to bonding failures on maxilla and mandible.

Bond failure occurred in 21 (92%) of all patients, and rebonding procedures used the same indirect bonding tray that has been used in orthodontic clinical practice.^1^ ^,^ ^14^ The most recurring bond failure occurred on mandibular second molars (50%), followed by maxillary second molars (45%), mandibular second premolars (28%), and maxillary first molars (15%). Two patients handled 23% of all the bond failures (11 and 8 rebondings for each of one, respectively) and had both mandibular second molars rebonded at least twice (Fig. 7). Two out of the 23 patients (9%) did not show any bond failure.

## DISCUSSION

Predicted occlusion outcome and treatment occlusion outcomes differs. The use of CAD-CAM technology to verify the accuracy and predictability between the ideal planned tooth position and the treated tooth position produced divergent results between the two methods. The present results showed that comparisons of the measurements between the Predicted and Treated groups presented significant differences in 53%, so the null hypothesis was rejected. The literature has shown that the accuracy of the different orthodontic systems differs depending on the type of software, tooth movement and position,^4^ and orthodontic biomechanics and knowledge of the orthodontists.^1^

### INTRAOBSERVER RELIABILITY

The first measurements were slightly larger than the second ones, but the systematic errors were smaller than 0.10 mm and 0.25°. Differences smaller than 0.20 mm and 1.00° have been suggested to be clinically insignificant.^11^ ^,^ ^15^ ^,^ ^16^ Importantly, intra-observer reliability ranged from 91% to 99%, showing high precision. Comparisons between both methods were highly reliable when all six measurements were evaluated for intraobserver reliability. The literature has shown that reliability coefficients above 0.75 are considered to be good to excellent.^1^ ^,^ ^17^ The present results are consistent with those of other authors who showed a high correlation based on 3D digital model measurements.^1^ ^,^ ^18^ ^-^ ^20^

### MAXILLARY LINEAR MEASUREMENTS

Linear measurements of the maxillary arch presented discrepancies between the virtual predicted group and the treated final occlusion group. The Treated group showed slightly larger distances (up to 2%) than the Predicted group, which imply in wider and longer maxillary arch form for the former group. Although some measurements were significantly different, clinically they may be acceptable, due to the differences being less than 0.70 mm, except for the arch perimeter being 1.77 mm (which is only approximately 2% larger). An example of clinical evaluation for treatment quality is the ABO-OGS score system, which subtracts points for incorrect alignment and leveling that deviate 0.50 mm or more from smaller observation measurements such as marginal ridge level.^5^ ^,^ ^13^ Moreira et al.^1^ showed significant differences only in 2 out of 8 ABO-CRE criteria (occlusal contacts and overjet). The present study employed the same samples used in that research, but using different methodologies, and showed threshold values above 86% in the linear dimensions of the samples between the Predicted and Treated groups. When both studies were confronted, despite using different methodologies with the same aim, the findings conflicted in relation to the predictability and accuracy of the CAD-CAM system.

### MAXILLARY TEETH ANGULATIONS AND INCLINATIONS

Maxillary teeth showed larger mesial crown tipping in the Treated group than in the Predicted group. Crown tipping differences were consistent in the anterior segment, increasing from the central incisor to the canine, and presenting similar values among posterior teeth, whereas the first premolar and the second molar showed differences between the two evaluated occlusions. Although mesiodistal angulation differences were seen between some teeth, the differences were smaller than 1.00° (second molar showed the largest difference). At the end of orthodontic treatment, inadequate angulation may cause a lack of occlusal relationship and inadequate tooth intercuspation. A crown-tip inadequacy angulation of 2.00° causes a discrepancy of 0.50 mm at marginal ridges in an average-sized molar.^11^ ^,^ ^13^ Despite methodological differences between this study and others,^1^ ^,^ ^6^ ^,^ ^7^ ^,^ ^11^ ^,^ ^13^ the findings were similar in relation to the limits considered clinically ideal (<2.00°) for maxillary central incisors, first premolars and second molars.^11^ Angulation of the accessory determines the correct axial position of teeth, contributing to a better and more stable position of teeth at the end of the treatment in their respective bone bases.

Teeth buccolingual inclination generally produces values above what is considered the ABO threshold. Central incisor, first premolar and second molar obtained the most discrepant values. In general, maxillary anterior teeth were more uprighted, and posterior teeth were more lingually inclined in the final occlusion than in the virtual predicted occlusion. In this study, maxillary anterior teeth, especially the central incisors of the Treated group, were more upright (12%), and the posterior teeth were slightly less (≤ 8%) inclined toward the palatal direction than those of the setup. It is important to mention that outliers were included, and an increase in size between the values ​​obtained was observed. It has been shown that CAD-CAM setup closely predicts the final buccolingual tooth inclination.^1^ It is very important to analyze the absolute values ​​as well as their magnitude, to judge whether these differences are clinically relevant or not. The literature has shown that orthodontic archwire bending robots provide predicted tooth position, but the effectiveness of the orthodontic treatment varies with tooth type and dimension of the movement.^4^ ^,^ ^11^ Previous report has shown that the maxillary central incisors were inclined less facially inclined and the posterior teeth were more facially inclined when robotic technology was used.^11^ It is possible that dental arch expansion obtained with the treatment required more torque expression to compensate for the wider and longer dental arch size, especially for the anterior segment. In addition, successive bonding failures, mainly of the second molar accessory, may have influenced the ideal bracket rebonding position and, consequently, the torque expression planned by the CAD-CAM system.^1^

### MANDIBULAR LINEAR MEASUREMENTS

Mandibular arch was wider and longer in the Treated group than in the Predicted group. Treated group arch presented larger linear differences (up to 3%) than the virtual arch. The largest difference (less than 2.00 mm) was found at the arch perimeter when both arches were compared, and this is clinically acceptable. Over the years, it has been proposed that mandibular intercanine width should be kept during the orthodontic treatment, and this study showed a difference smaller than 0.50 mm between the two evaluated groups. Although the literature has shown minimal or no difference between digitally created 3D models,^6^ ^,^ ^20^ ^,^ ^21^ the Treated group was evaluated from a cast model that was scanned, and there was a risk of dimensional deformation due to traditional alginate impression. Vestibular inclination of mandibular central incisors (0.63 mm) may explain the lengthening of the perimeter. 

### MANDIBULAR TEETH ANGULATIONS AND INCLINATIONS

Mandibular mesiodistal crown angulation and mandibular buccolingual crown inclination differed between Predicted and Treated groups. In 43% of the angular measurements, there were differences between the virtual setup and final tooth position. Interesting is the fact that there was no pattern for the angular differences when the virtual setup and treated teeth positions were evaluated. Overall, mandibular teeth presented acceptable buccolingual differences between both groups, with the largest exception being the buccolingual crown inclination of the second molar (approximately 10.00°), followed by the first molar and second premolar.

Posterior teeth angular measurements exceeded the threshold limits reported in a previous study.^11^ The literature has reported different assessments of buccolingual crown inclination in orthodontic planning and treatment systems using the ABO Grading System scores.^1^ ^,^ ^7^ ^,^ ^13^ However, since the guidelines did not score the mandibular first premolars or the distal cusps of the second molars, there could be a bias in the interpretation of the findings. The Treated group produced comparable torque values with those reported by other studies using tridimensional software.^22^ ^-^ ^24^ Literature has also shown that bracket placement differs between experienced orthodontists and bonding technique.^25^ In addition, it is important to emphasize that the mandibular second molar presented the highest rebonding rate of all teeth, which may influence the programed bracket bonding reposition and torque expression intended by the CAD-CAM system (Fig. 6). 

### EFFECTIVENESS OF THE CAD-CAM TECHNOLOGY

Discrepancies between the virtual treatment occlusion and the treated occlusion outcomes cannot be attributed completely to a lack of effectiveness of the CAD-CAM technology. To produce the planned individual arch shape and tooth position prescribed by the virtual treatment plan, numerous variables can influence the orthodontic treatment efficiency and effectiveness. Tooth crown, root anatomy, periodontal condition, bone density, and/or occlusal forces may prevent a tooth from moving exactly as planned.^26^ Furthermore, the number of broken accessories and consequent bracket rebonding can influence the discrepancies between virtual setup and treated occlusions. In addition, some of the angular differences can be attributed to insufficient torque expression due to torque play between archwire cross-section and slot size.^27^ Importantly, the present findings did not suggest an unsatisfactory treatment outcome. Previous studies showed that CAD-CAM technology combined with the indirect bonding method was an essential method to achieve excellence in orthodontic treatment.^1^ ^,^ ^28^ It is important to highlight that the final occlusion result was measured before intermaxillary elastics and wire bends. Therefore, occlusal contact areas were expected to increase during the retention phase.^1^ A recent study using ABO-CRE scores showed that CAD-CAM system setup closely predicted the final teeth alignment and leveling, possibly improving root parallelism after treatment.^1^ The present study used the same samples of in the aforementioned research, but used a different methodology. When comparing both study outcomes, the present findings suggest that ABO-CRE may be less accurate, when compared to crown linear and angular measures. 

### LIMITATIONS

This study was designed using mild malocclusions to limit and standardize dental movements within an expected pattern. However, in the orthodontic clinic, malocclusions are often the result of different combinations of arch space, tooth anatomy and size, and type of growth pattern. Moreover, bone density and bone growth factors, periodontal restrictions, root morphology of the teeth, and a lack of patient cooperation can also induce discrepancies between the predicted and the actual treatment outcome.^4^ ^,^ ^29^ In addition, this study did not evaluate occlusion outcome. Therefore, due to these limitations, further studies are recommended to apply this methodology to evaluate the predictability of the CAD-CAM system in different types of malocclusions. Undoubtedly, the use of artificial intelligence in different CAD-CAM systems is growing, and it has helped to improve the efficiency of orthodontic treatment.^30^ Digital setups help orthodontists to refine aspects of alignment and leveling, arch form, torque, and occlusal contacts even before the treatment begins.

The superimposition method adopted in this study using predicted and treated occlusion models with geometric reference planes enabled consistent results related to tooth positioning changes. Although the Geomagic software algorithm can perform the superimposition of the Predicted and Treated arch forms efficiently, it may not have been enough to obtain differences in tooth position based only on the best fit method. In addition, the iterative method of the closest point algorithm, which has been used in different studies, may be a source of bias in the outcomes.^29^ Because the 3D models of the predicted group were digitally modified by eXceed software to obtain the occlusion, they did not have the palatal rugae. For this reason, using a novel superimposition methodology, teeth were used instead of soft tissue to record the coincident points and obtain the superimposition of the 3D models. Some strategies were applied in 3D models to reduce the bias of the lack of stable anatomical points, such as mesh refinement prior to applying superposition method, to enrich the mesh by reducing the size of the elements and improving mesh quality. The results obtained in the present study recommend future research to compare measurements of tooth angulations using stable anatomical reference points with the method used in this study. In addition, with the advent of new digital methods for clinicians, further studies are recommended to verify the consistence and effectiveness in orthodontic treatment using digital software systems.

## CONCLUSIONS

Measures from Predicted and Treated groups obtained from Angle Class I malocclusion subjects, with no required orthodontic extractions, produced accurate and reliable representations. Based on measurement comparisons between the two groups, the following conclusions can be drawn:

1. The Treated group had wider and longer maxillary and mandibular arches than the Predicted group. The arch perimeter showed the greatest inaccuracies of the linear variables.
2. Maxillary teeth were more upright in the anterior segment and more lingually inclined in the posterior segments in the Treated group than in the Predicted group.
3. Maxillary teeth presented larger mesial crown tipping in the Treated group than in the Predicted group.
4. Mesiodistal crown angulation was the most accurate in both maxillary and mandibular arches.
5. Buccolingual crown inclination obtained the most values ​​above the ABO threshold, when compared to all parameters analyzed.
6. Indirect bonding programmed by the orthodontic systems helps clinicians in the treatment of dental malocclusions.
7. The digital system analyzed did not show accuracy in torque predictions in clinical cases, requiring knowledge and skills of orthodontists.
